# Crustose lichens with lichenicolous fungi from Paleogene amber

**DOI:** 10.1038/s41598-019-46692-w

**Published:** 2019-07-17

**Authors:** Ulla Kaasalainen, Martin Kukwa, Jouko Rikkinen, Alexander R. Schmidt

**Affiliations:** 1 Department of Geobiology, University of Göttingen, Goldschmidtstraβe 3, 37077 Göttingen, Germany; 2 Department of Plant Taxonomy and Nature Conservation, Faculty of Biology, University of Gdańsk, Wita Stwosza 59, 80-308 Gdańsk, Poland; 3 Finnish Museum of Natural History, P.O Box 7, 00014 University of Helsinki, Helsinki, Finland; 4 Organismal and Evolutionary Biology Research Programme, Faculty of Biological and Environmental Sciences, P.O Box 65, 00014 University of Helsinki, Helsinki, Finland

**Keywords:** Palaeontology, Fungal evolution, Taxonomy, Palaeoecology, Coevolution

## Abstract

Lichens, symbiotic consortia of lichen-forming fungi and their photosynthetic partners have long had an extremely poor fossil record. However, recently over 150 new lichens were identified from European Paleogene amber and here we analyse crustose lichens from the new material. Three fossil lichens belong to the extant genus *Ochrolechia* (Ochrolechiaceae, Lecanoromycetes) and one fossil has conidiomata similar to those produced by modern fungi of the order Arthoniales (Arthoniomycetes). Intriguingly, two fossil *Ochrolechia* specimens host lichenicolous fungi of the genus *Lichenostigma* (Lichenostigmatales, Arthoniomycetes). This confirms that both *Ochrolechia* and *Lichenostigma* already diversified in the Paleogene and demonstrates that also the specific association between the fungi had evolved by then. The new fossils provide a minimum age constraint for both genera at 34 million years (uppermost Eocene).

## Introduction

Lichens are highly specialized mutualistic symbioses, in which a dominant fungal symbiont (mycobiont) hosts one or several taxa of phototrophic green algae and/or cyanobacteria (photobionts). The vast majority of the over 19 500 currently known species of lichen-forming fungi belong to the Ascomycota^1,2^. Many of them grow as tightly adhered crusts on their substrate, mostly on rock, soil, or bark. Crustose lichens are found in almost all major terrestrial biomes ranging from the tropics to polar regions.

In addition to the myco- and photobionts, lichen thalli commonly support diverse assemblages of associated microfungi and bacteria^3–5^. Lichenicolous fungi are a diverse group of obligate associates of lichens. A vast majority of them are ascomycetes, but the group also includes taxa from several classes of the basidiomycetes^6,7^. The specificity of different lichenicolous associations varies from general to highly specific^6^^,8^. Highly specific associations often involve specialized infection structures and exhibit relatively low virulence^6^.

Succinite is the major variety of amber (fossil resin) from large Paleogene European deposits located in the Baltic area (Baltic states, Poland, western Russia and adjacent regions) and near the city of Bitterfeld in central Germany. Succinite was recently shown to preserve numerous relatively well-preserved lichen fossils^9^, multiplying the known fossil record of lichens over tenfold. Regardless of the sometimes exceptional preservation of the amber inclusions and the utilization of modern research methods, a reliable identification of even the larger foliose and fruticose lichen inclusions is very challenging^10,11^. The anatomy of fossils preserved in amber can only rarely be studied, and information on many crucial characters such as spore size and septation, ascoma structure, and cortex type are rarely available. In many extant lichen lineages, lichen secondary chemistry provides important clues for distinguishing between taxa^12^ that cannot be examined from amber inclusions. Due to these limitations, only four extant lichen genera have unambiguously been identified from amber specimens so far, namely *Anzia* Stizenb. (Parmeliaceae), *Calicium* Pers. (Caliciaceae), and *Chaenotheca* (Th. Fr.) Th. Fr. (Coniocybaceae) from Paleogene European amber^13–16^, and *Phyllopsora* Müll. Arg. (Ramalinaceae) from Miocene Dominican amber^17,18^. Confidently assigned fossils provide minimum ages for the respective lineages and represent the standard for the calibration of divergence time estimations^19^. The still few confidently assigned lichen fossils have significantly deepened our understanding in the origins and evolution of the various lineages of Ascomycota^15,20‒22^. Fossil evidence of interactions between microfungi and lichens have so far been limited to more general and likely saprotrophic associations of filamentous microfungi and decomposing lichen thalli^23,24^.

Most crustose lichens are relatively small and detaching them from the substrate is often impossible without major damage to the thallus. This has hindered the preservation and detection of crustose lichens in amber in two ways: firstly, large pieces of lichen substrate are rarely preserved, and secondly, any preserved fossils go easily undetected. For these reasons, with the exception of calicioid lichens, crustose species were until very recently absent from the fossil record. However, our recent survey demonstrated that crustose lichens are indeed present in European Paleogene amber^9^. In this study we analyse the most spectacular examples of fossil crustose lichens and also elucidate their associations with lichenicolous fungi.

## Results

**Figure 1:**
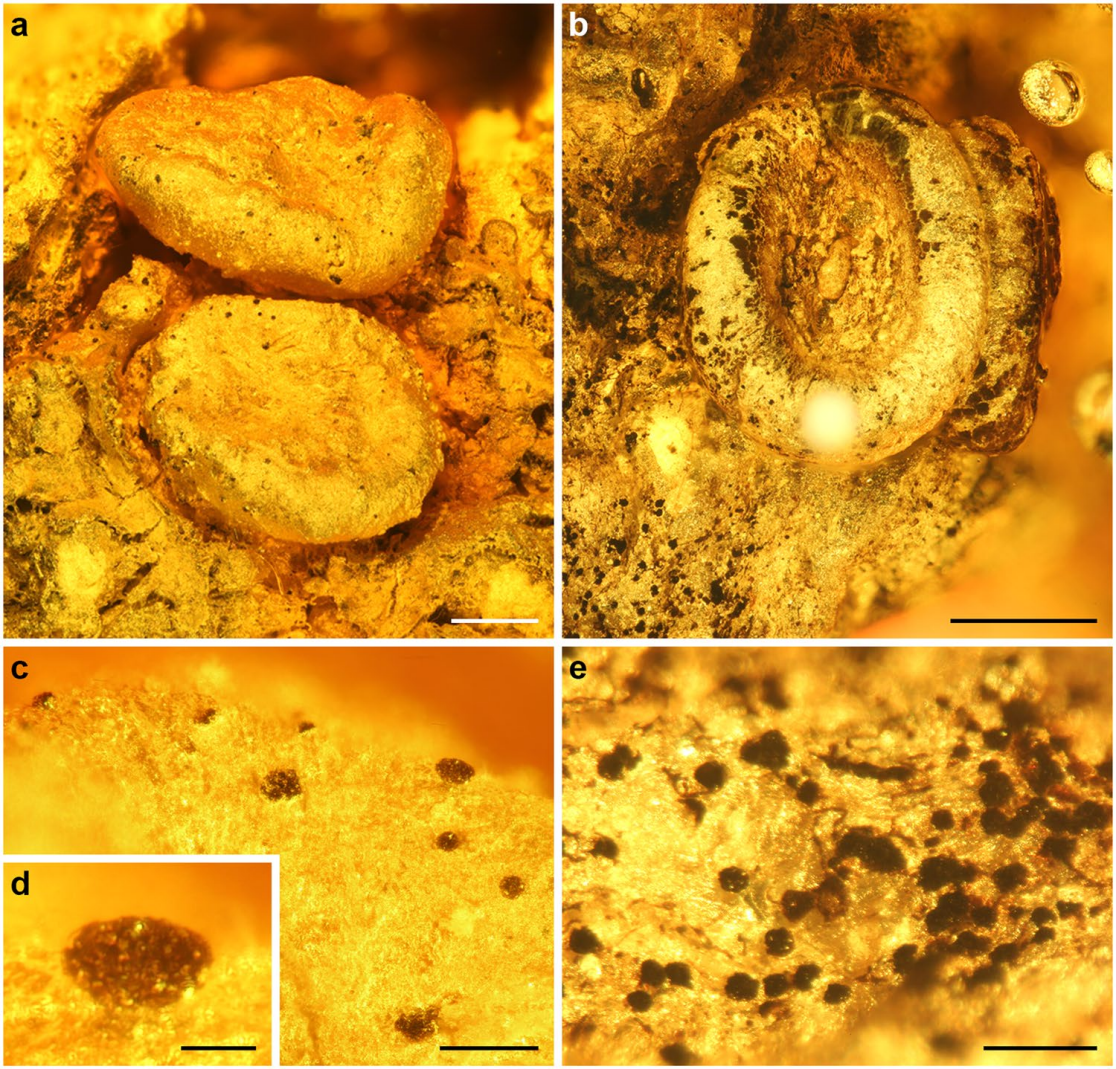
Fossil *Ochrolechia* specimens with lichenicolous *Lichenostigma* from European Paleogene amber. (**a**) *Ochrolechia* with apothecia, growing together with a foliose lichen (GZG.BST.21924). The black dots on the apothecial margin (**c**,**d**) are conidiomata and/or ascomata of the lichenicolous fungi *Lichenostigma*. (**b**) *Ochrolechia* with apothecia and (**e**) *Lichenostigma* on the crustose thallus (GZG.BST.27293). Scale bars 500 µm in (**a**,**b**), 100 µm in (**c**,**e**), 20 µm in (**d**).

**Figure 2:**
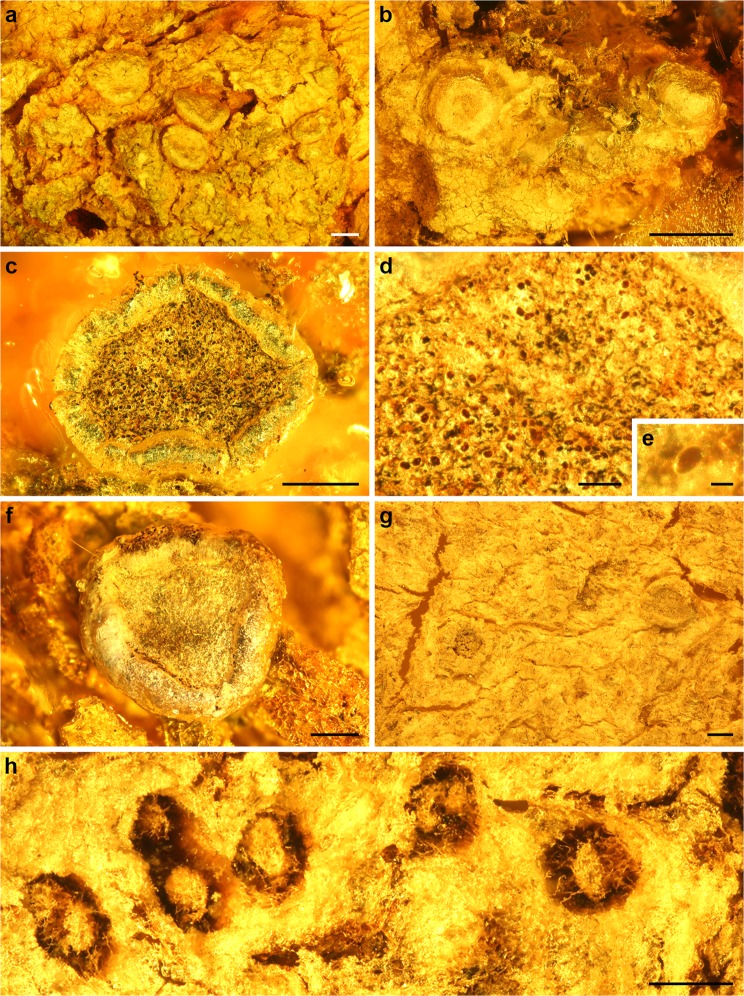
Crustose lichens from European Paleogene amber. *Ochrolechia* (**a**,**b**), and unidentified crustose lichens (**c**,**d**). (**a**,**b**) *Ochrolechia* with apothecia, growing together with foliose lichens. (**c**) Apothecium. (**d**) Ascospores on the upper surface of the apothecial disk. (**e**) Unicellular ascospore. (**f**,**g**) Apothecia. (**h**) Specimen of Arthoniales with byssoid thallus and conidiomata. The optical sections of immersed conidiomata are seen from the lower side and show hyphae and masses of conidia preserved within the conidiomata. (**a**) GZG.BST.21924, (**b**) GZG.BST.27298, (**c**–**e**) GZG.BST.21982, (**f**) GZG.BST.21981, (**g**) GZG.BST.21915, and (**h**) GZG.BST.21925. Scale bars 1 mm in (**a**,**b**), 200 µm in (**c**,**f**–**h**), 50 µm in (**d**), and 10 µm in (**e**).

All studied fossils are fertile and represent lichen-forming species of the Ascomycota, preserved on degraded bark remains inside amber. Three fossils belong to the genus *Ochrolechia* A. Massal. (Ochrolechiaceae, Pertusariales; Figs 1a,b and 2a,b) and one is assigned to the order Arthoniales (Fig. 2h). Additionally, conidiomata and/or ascomata of the lichenicolous fungi *Lichenostigma* Hafellner (Lichenostigmatales; Fig. 1c–e) are preserved on two *Ochrolechia* specimens. Nine additional fossils represent crustose lichens which cannot with confidence be assigned to extant genera. For the detailed description of each fossil, see Supplementary material.

### Systematic Paleontology

Division Ascomycota Cavalier-Smith, 1998

Subdivision Pezizomycotina O.E. Erikss. & Winka, 1997

Class Lecanoromycetes O.E. Erikss. & Winka, 1997

Subclass Ostropomycetidae Reeb, Lutzoni & Cl. Roux, 2004

Order Pertusariales M. Choisy ex D. Hawksw. & O.E. Erikss.

Family Ochrolechiaceae R.C. Harris ex Lumbsch & I. Schmitt, 2006

Genus *Ochrolechia* A. Massal., 1852

Description: Crustose lichens with apothecia (Figs 1a,b and 2a,b). Crustose thallus thin, vegetative diaspores not present. Apothecia sessile, large (0.9–2.0 mm in diameter), with prominent, smooth and even margins. Apothecial discs even or concave, pruina not visible.

Specimens examined: Collections of the Geoscience Centre at the University of Göttingen GZG.BST.21924, GZG.BST.27293, and GZG.BST.27298.

Remarks: Conidiomata and/or ascomata of the lichenicolous fungus *Lichenostigma* are present on the apothecial margin and crustose thallus of two specimens (Fig. 1c–e).

Class Lecanoromycetes O.E. Erikss. & Winka, 1997

Subclass, family & genus incertae sedis

Description: Corticolous crustose lichens with apothecia (Fig. 2c,f). Crustose thallus not visible. Apothecia sessile, irregular and angular in shape, 0.7–0.9 mm in diameter, with prominent, smooth to crenate and uneven margins. Apothecial discs even and possibly covered by thin pruina. Ascospores simple, elliptical, brown, and 15 × 9 µm in size (Fig. 2d,e; based on specimen GZG.BST.21982).

Specimens examined: Collections of the Geoscience Centre at the University of Göttingen GZG.BST.21981 and GZG.BST.21982.

Class Lecanoromycetes O.E. Erikss. & Winka, 1997

Subclass, order, family & genus incertae sedis

Description: Corticolous crustose lichens with apothecia (an example in Fig. 2g). Crustose thallus thin, degraded, or not visible. Vegetative diaspores rarely present (possible soredia present in one specimen). Apothecia sessile or rarely partially immersed in bark, round to oval, 0.1–0.7 mm in diameter. Apothecial margins (when observable) prominent to existent, often smooth and even. Apothecial discs (when observable) even and possibly covered with thin pruina.

Specimens examined: Collections of the Geoscience Centre at the University of Göttingen GZG.BST.21915, GZG.BST.21930, GZG.BST.21941, GZG.BST.21942, GZG.BST.21983, GZG.BST.21984, and GZG.BST.21985.

Remarks: The specimens may represent several taxa. However, the preservation does not allow their assignment to extant genera. On the crustose thallus of two specimens, cell-chains and/or conidiomata/ascomata of possibly lichenicolous fungi are present (Fig. S1).

Class Arthoniomycetes O.E. Erikss. & Winka, 1997

Order Arthoniales Henssen ex D. Hawksw. & O.E. Erikss., 1986

Family and genus incertae sedis

Description: Corticolous crustose lichen with a byssoid thallus and numerous conidiomata (Fig. 2h). Conidiomata dark, slightly oval and ca. 0.2 mm in diameter, filled with light masses of conidia.

Specimens examined: Collections of the Geoscience Centre at the University of Göttingen GZG.BST.21925

Class Arthoniomycetes O.E. Erikss. & Winka, 1997

Order Lichenostigmatales Ertz, Diederich & Lawrey, 2014

Family Phaeococcomycetaceae McGinnis & Schell

Genus *Lichenostigma* Hafellner, 1983

Description: Lichenicolous fungi growing on crustose lichen thalli and apothecial margin (Fig. 1c–e). Conidiomata and/or ascomata up to 40 µm in diameter, consisting of subglobular cells of 2–5 µm size.

Specimens examined: Collections of the Geoscience Centre at the University of Göttingen GZG.BST.21924 and GZG.BST.27293.

Remarks: *Lichenostigma* is found growing on two *Ochrolechia* specimens. Conidiomata and ascomata of *Lichenostigma* are morphologically indistinguishable.

## Discussion

Several studies on Ascomycota have recently utilized fossil fungi for the calibration of evolutionary analyses^15,20,22^. However, among the lichen-forming genera only the Paleogene *Anzia* (Parmeliaceae, Lecanoromycetes), *Calicium* (Caliciaceae, Lecanoromycetes), and *Chaenotheca* (Coniocybaceae, Coniocybomycetes) and Miocene *Phyllopsora* (Ramalinaceae, Lecanoromycetes) have been available to add confident minimum age constraints for lichenized and lichen-associated fungal genera. Identification of crustose lichens is very challenging even with extant specimens, and often without precise information on anatomy (e.g. ascus structure, apothecial margin anatomy or size and septation of ascospores) and the secondary chemistry (acetone-soluble lichen metabolites or insoluble pigments)^25,26^ even assignment to higher taxonomic categories is impossible. In this study we present fossils that can be used as new genus-level calibration points for *Ochrolechia* and *Lichenostigma* within the Pertusariales and the Lichenostigmatales by setting the minimum age of both genera to 34 million years.

*Ochrolechia* is a genus of mostly corticolous or saxicolous crustose lichens with relatively large and conspicuous apothecia with prominent margins. Of the studied fossil amber inclusions, several well-preserved specimens were identified as *Ochrolechia* based on general habit and a combination of morphological characters. The large, round to slightly oval apothecia with thick and prominent margins, together with the shape of the disc and the attachment of the apothecial exciple to the apothecial disc place the fossil specimens within *Ochrolechia*. Close morphological equivalents exist among extant *Ochrolechia* species: for example, specimen GZG.BST.21924 has large apothecia 1‒2 mm in diameter with prominent and even margins, and smooth discs (Figs 1a and 2a), very similar to *O*. *subplicans* (Nyl.) Brodo and *O*. *xanthostoma* (Sommerf.) K. Schmitz & Lumbsch while specimen GZG.BST.27293 has sessile, slightly oval, approximately 1.3 mm in diameter apothecia with very prominent, smooth, and even margins, and clearly concave discs (Fig. 1b), closely resembling *O*. *balcanica* Verseghy^27,28^.

In addition to DNA characters, the identification of the approximately 60 extant *Ochrolechia* species relies largely on secondary chemistry, but also the shape, size, and margin type of the apothecia and the presence of pruina are important for species determination^28–34^. In some extreme cases, only the geographical distribution, differences in the specific placement of lichen substances within thalli, or other subtle differences in lichen chemistry distinguish between morphologically cryptic, but genetically distinct species^34,35^. Because of obvious limitations in the biochemical analysis of amber-preserved lichens, we cannot determine if the fossil *Ochrolechia* specimens represent one or several species.

The distinctly smaller size of the apothecia of the well-preserved fossils within amber specimen GZG.BST.21915 (Fig. 2g) and GZG.BST.21941 distinguish them from the *Ochrolechia* specimens. Additionally, the apothecial margins and general morphology of the apothecia resembles those of for example some extant *Lecanora* Ach. species. Amber specimens GZG.BST.21981 (Fig. 2f) and GZG.BST.21982 (Fig. 2c–e) contain well-preserved inclusions, and in the latter amber has even captured the ascospores that are in the process of being released from the apothecium (Fig. 2d). However, despite these interesting features, the fossils cannot be assigned to any one genus.

The fossil crustose lichen with a byssoid thallus and dark conidiomata resembles some extant taxa of the Arthoniales. This order includes five to six families of crustose and fruticose lichens^36^. The conidiomata of the fossil closely resemble those of some *Inoderma* and *Lecanactis* species^37,38^. However, the fossil cannot be assigned to any one genus, especially as it has recently been shown that the traditional morphology-based circumscription of taxa within Arthoniales does not correspond with gene-derived phylogenies^36,39–41^. The unusually high level of homoplasy indicate that Arthoniales is an ancient group of fungi and that the immediate precursors of some genera may have already existed in the Mesozoic^36,42^.

The genus *Lichenostigma* currently contains five lichenicolous species^8,43^, of which *L*. *alpinum* (R. Sant., Alstrup & D. Hawksw.) Ertz & Diederich is known from several extant species of *Ochrolechia*^43^. The conidiomata and/or ascomata of the extant *Lichenostigma* species are similarly sized and composed of identical subspherical cells as the fossil *Lichenostigma*. In addition to the *Ochrolechia* specimens, microfungi are present also in two other crustose lichen inclusions. The appearance of the fungi is similar to *Lichenostigma* but in addition to the conidiomata/ascomata, also moniliform hyphae is present (Fig. S1). Among the extant *Lichenostigma*, presence of mycelium is extremely rare, and, especially since we are lacking more detailed information about the host taxa of these fungi, it is possible that they rather represent some other taxa for example within *Lichenostigma* s. lat.^43^.

Previous studies have not been successful in estimating the divergence time of the family Ochrolechiaceae with any precision, with estimates spanning from the Late Cretaceous to the Paleogene^20^, and no previous divergence time estimates are available for Lichenostigmataceae. Our new findings show that both *Ochrolechia* and *Lichenostigma* were already present 34 million years ago, i.e., in uppermost Eocene for which Baltic amber provides a preservation window. In contrast to all lichen-associated filamentous microfungi previously described from European Paleogene amber^23,24^, *Lichenostigma* represents a true mycoparasite, providing the first fossil evidence of these highly specialized and ecologically important associations. It also demonstrates that the intimate link between *Lichenostigma* and its hosts is ancient and most probably traces back to the Mesozoic.

## Materials and Methods

The studied fungal fossils (Table 1) are part of the collections of the Geoscience Centre at the University of Göttingen (GZG). Seven of the fossils are preserved in Baltic amber and six in Bitterfeld amber. Detailed descriptions of each specimen are included in the Supplementary material.

**Table 1:** Specimens of fossil crustose lichens and fungi examined in this study.

Fossil	Collection number	Former collection number	Source	Illustration
*Ochrolechia* sp., *Lichenostigma*	GZG.BST.21924	Hoffeins Amber Collection 1069-1	Baltic amber	1a, c & d, 2a
*Ochrolechia* sp., *Lichenostigma*	GZG.BST.27293	Heinrich Grabenhorst Amber Collection Li-3	Bitterfeld amber	1b & e
*Ochrolechia* sp.	GZG.BST.27298	Heinrich Grabenhorst Amber Collection Li-54	Bitterfeld amber	2b
Crustose lichen, lichenicolous fungi	GZG.BST.21984	Heinrich Grabenhorst Amber Collection Ri-35	Baltic amber	
Crustose lichen, lichenicolous fungi	GZG.BST.21985	Heinrich Grabenhorst Amber Collection Ri-51	Baltic amber	S1
Arthoniales (cf.)	GZG.BST.21925	Hoffeins Amber Collection 1069-4	Baltic amber	2h
Crustose lichen	GZG.BST.21983	Heinrich Grabenhorst Amber Collection Ri-20	Bitterfeld amber	
Crustose lichen	GZG.BST.21915	Hoffeins Amber Collection 1040-15	Baltic amber	2g
Apothecium	GZG.BST.21981	Heinrich Grabenhorst Amber Collection Li-17	Bitterfeld amber	2f
Apothecia	GZG.BST.21982	Heinrich Grabenhorst Amber Collection Li-19	Bitterfeld amber	2c–e
Degraded crustose lichen	GZG.BST.21941	Hoffeins Amber Collection 88-3	Bitterfeld amber	
Degraded apothecia	GZG.BST.21930	Hoffeins Amber Collection 72-1	Baltic amber	
Degraded apothecia	GZG.BST.21942	Hoffeins Amber Collection 968-3	Baltic amber	

The Eocene sediments containing Baltic amber are 34–47 million years old^44,45^. Baltic amber primarily derives from the marine Blue Earth layer that is mainly mined on the Samland Peninsula northwest of Kaliningrad (Russia). Baltic amber eroded from sediments is abundantly found washed ashore along the coast of the Baltic Sea^45,46^. The absolute age of Baltic amber is still under debate. Palynological data suggest an upper Eocene (Priabonian) age (ca. 38–34 Ma) of the Blue Earth but fewer amounts of Baltic amber also occur in Lutetian (middle Eocene) sediments^44,45,47^.

Bitterfeld amber originates from the Goitzsche mine near the city of Bitterfeld in central Germany. It occurs in the Chattian ‘Bernsteinschluff’ Horizon in the upper part of the Cottbus Formation. The Upper Oligocene amber-bearing sediment has an absolute age of 23.8–25.3 million years^48,49^. A notion that Bitterfeld amber represents redeposited Eocene Baltic amber is based on the fact that there is a significant proportion of identical arthropod morphologies in amber from both localities^50^. However, redeposition of Baltic amber is unlikely, based on the reconstruction of the sedimentary environment of this large amber deposit^45^. A local reworking of pre-Chattian amber, however, has not been dispelled so far^51^. In any case, Bitterfeld amber is Paleogene in age and its minimum age is ca. 24 Ma.

The amber pieces were ground and polished manually using a series of wet silicon carbide papers (grit from FEPA P 600–4000, Struers, Germany) to produce smooth amber surfaces as close to the fossil inclusions as possible to minimize light distortion for imaging but still ensuring the preservation of the fossil. The fossils were examined under a Carl Zeiss Stereo Discovery V8 dissecting microscope and a Carl Zeiss AxioScope A1 compound microscope, each equipped with a Canon EOS 5D digital camera. In most cases, incident and transmitted light were used simultaneously. The images are digitally stacked photomicrographic composites obtained from up to 130 focal planes using the software package Helicon Focus (Version 6.3.3 Pro).

## Supplementary information

Description of the fossil specimens

## Data Availability

All specimens are part of the public collection of the Geoscience Centre at the University of Göttingen.
