# Supplementary material for: Crustose lichens with lichenicolous fungi from Paleogene amber
Source: Sci Rep. 2019 Jul 17;9:10360. doi: 10.1038/s41598-019-46692-w (PMC6637111; doi:10.1038/s41598-019-46692-w)
Supplement: Supplementary file 1 — Description of the fossil specimens [file 41598_2019_46692_MOESM1_ESM.pdf]

Scientific Reports, Supplementary material

Crustose lichens with lichenicolous fungi from Paleogene amber

Ulla Kaasalainen[1]*, Martin Kukwa[2], Jouko Rikkinen[3, 4], and Alexander R. Schmidt[1]

[1] Department of Geobiology, University of Göttingen, Goldschmidtstraβe 3, 37077 Göttingen, Germany

[2] Department of Plant Taxonomy and Nature Conservation, Faculty of Biology, University of Gdańsk, Wita Stwosza 59, 80-308 Gdańsk, Poland

[3] Finnish Museum of Natural History, P.O Box 7, 00014 University of Helsinki, Finland

[4] Organismal and Evolutionary Biology Research Programme, Faculty of Biological and Environmental Sciences, P.O Box 65, 00014 University of Helsinki, Finland

* Corresponding author: ulla.kaasalainen@uni-goettingen.de

Description of the fossil specimens

Specimen GZG.BST.21915 (Hoffeins Amber Collection 1040-15), Fig. 2g
Fossil lichen with many apothecia (> 20) widely distributed over bark remains. Crustose thallus very thin, without isidia or soredia. Apothecia sessile and circular, c. 0.4 mm in diameter. Apothecial margins prominent, smooth to rugose (possibly due to imperfect preservation), or even. Discs even, possibly covered by thin pruina.

Specimen GZG.BST.21924 (Hoffeins Amber Collection 1069-1), Figs 1a, c, d, 2a
Identification: *Ochrolechia* sp. with *Lichenostigma* sp. Fossil lichen with six apothecia on bark. Crustose thallus thin, isidia and soredia absent. Five apothecia are well preserved: they are sessile, slightly angular, 1.0–2.0 mm in diameter, margins prominent, more or less smooth and even. Discs appear smooth and lack pruina. The apothecial margins have small black dots representing conidiomata and/or ascomata of the lichenicolous fungus *Lichenostigma*. The conidiomata/ascomata are up to approximately 40 µm in diameter and consist of subglobular cells (Fig. 1c–d).

Specimen GZG.BST.21925 (Hoffeins Amber Collection 1069-4), Fig. 2h
Identification: Unknown genus in Arthoniales. Crustose lichen with a byssoid thallus and numerous conidiomata (sporodochia or pycnidia). The dark, slightly oval conidiomata are ca. 0.2 mm in diameter, and have a light central part corresponding with masses of conidia produced inside the conidiomata.

Specimen GZG.BST.21930 (Hoffeins Amber Collection 72-1)
Three apothecia on substratum. Thallus not visible. Apothecia round to oval, approximately 0.5 mm in diameter. Preservation poor with the inclusion largely hidden by fractures in amber matrix.

Specimen GZG.BST.21941 (Hoffeins Amber Collection 88-3)
Four poorly preserved apothecia. Thallus not visible. Apothecia sessile and round, 0.14–0.44 mm in diameter. Apothecial margins smooth and even. Discs hardly visible.

Specimen GZG.BST.21942 (Hoffeins Amber Collection 968-3)
Single apothecium partly immersed within bark, also some imprints of other apothecia visible. Thallus not visible. Apothecium round, 0.54 mm in diameter. Structure badly deteriorated before preservation with no details of margin or disc preserved.

Specimen GZG.BST.21981 (Heinrich Grabenhorst Amber Collection Li-17), Fig. 2f
Four apothecia, one of which is especially well preserved. Crustose thallus not visible, isidia and soredia absent. Apothecia sessile and irregular in shape, up to 0.88 mm in diameter. Apothecial margins prominent, smooth and uneven. Discs even, possibly covered by thin pruina.

Specimen GZG.BST.21982 (Heinrich Grabenhorst Amber Collection Li-19), Fig. 2c–e
One well preserved apothecium. Crustose thallus poorly visible, without isidia or soredia. Apothecium sessile and irregular in shape, 0.7 mm in diameter. Apothecial margin prominent, smooth to crenate. Discs even, possibly covered by thin pruina. Spores simple, elliptical, brown, and 15 x 9 µm in size (Fig. 2d–e).

Specimen GZG.BST.21983 (Heinrich Grabenhorst Amber Collection Ri-20)
Two apothecia on bark. Thallus not visible. Apothecia sessile and round, 0.42 mm and 0.6 mm in diameter. Apothecial margins prominent, smooth and even. Discs not visible. General habit resembles that of *Ochrolechia*, but the apothecia are small and their preservation poor.

Specimen GZG.BST.21984 (Heinrich Grabenhorst Amber Collection Ri-35)
Crustose lichen with apothecia. Crustose thallus degraded. Apothecia sessile, round, approximately 0.7 mm in diameter, not well preserved. Together with a putative lichenicolous fungus with conidiomata/ascomata with subglobular cells.

Specimen GZG.BST.21985 (Heinrich Grabenhorst Amber Collection Ri-51), Fig. S1
Crustose lichen with apothecia. Crustose thallus degraded, possibly with some soredia. Apothecia round, 0.22–0.3 mm in diameter, sessile, not well-preserved. Together with a putative lichenicolous fungus with cell-chains of spherical, regular cells of approximately 2–3 µm in diameter and conidiomata/ascomata with subglobular cells 2–5 µm in diameter (Fig. S1).

Specimen GZG.BST.27293 (Heinrich Grabenhorst Amber Collection Li-3), Figs 1b, e
Identification: *Ochrolechia* sp. with *Lichenostigma* sp. Fossil lichen with two well preserved apothecia. Crustose thallus highly degraded, vegetative diaspores not present. Apothecia sessile, slightly oval, diameter, approximately 1.3 mm, with prominent, smooth, and even margins. Discs scabrid and lacking pruina. Numerous small black dots, few present also on the apothecial margin but especially near the apothecia, representing conidiomata and/or ascomata of the lichenicolous fungus *Lichenostigma*. Conidiomata/ascomata (10–)20–40 µm in diameter, constructed of subglobular cells (Fig. 1e).

Specimen GZG.BST.27298 (Heinrich Grabenhorst Amber Collection Li-54), Fig. 2b
Identification: *Ochrolechia* sp. Fossil lichen with three relatively well-preserved apothecia. Crustose thallus thin, isidia and soredia absent. Apothecia sessile, c. 0.9 mm in diameter, with prominent, even and smooth margins. Discs even, with no pruina.

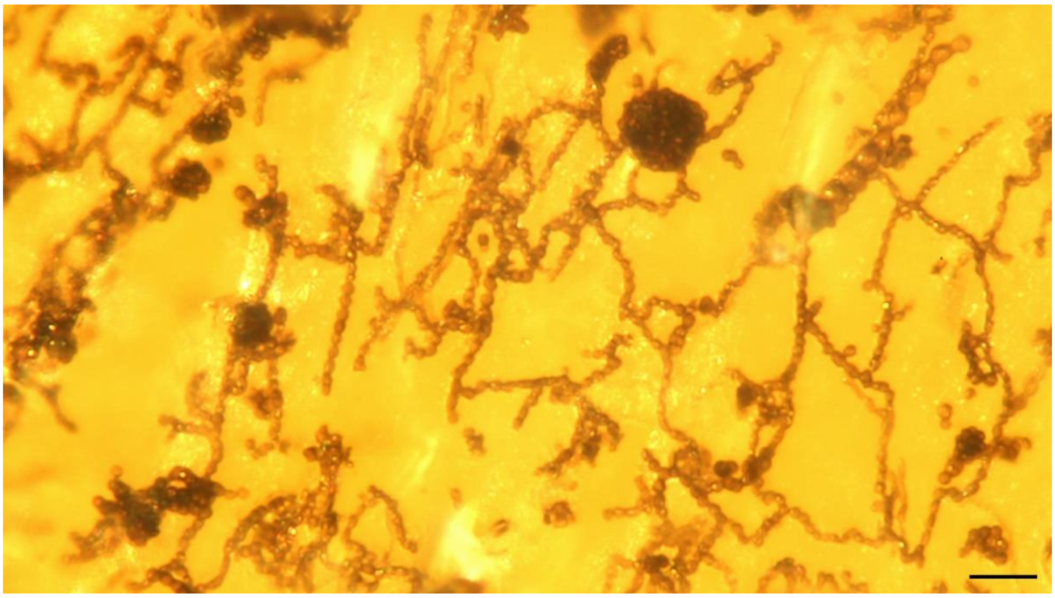

Figure S1. Branched moniliform hyphae and conidiomata/ascomata of a putative lichenicolous fungus (GZG.BST.21985). The fragment of larger moniliform hypha on the upper right belongs to a sooty mould (Capnodiales)[1]. Scale bar 20 µm.

References

1. Schmidt, A. R. *et al.* Amber fossils of sooty moulds. *Rev. Palaeobot. Palynol.* **200**, 53–64 (2014).
